# The dynamics of early-state transcriptional changes and aggregate formation in a Huntington’s disease cell model

**DOI:** 10.1186/s12864-017-3745-z

**Published:** 2017-05-12

**Authors:** Martijn van Hagen, Diewertje G. E. Piebes, Wim C. de Leeuw, Ilona M. Vuist, Willeke M. C. van Roon-Mom, Perry D. Moerland, Pernette J. Verschure

**Affiliations:** 1 Synthetic, Systems Biology and Nuclear Organization, Swammerdam Institute for Life Sciences, University of Amsterdam, Amsterdam, The Netherlands; 2 Bioinformatics Laboratory, Department of Clinical Epidemiology, Biostatistics and Bioinformatics, Academic Medical Center, University of Amsterdam, Amsterdam, The Netherlands; 3 MicroArray Department, University of Amsterdam, Amsterdam, The Netherlands; 4 Department of Human Genetics, Leiden University Medical Center, Leiden, The Netherlands

**Keywords:** Huntington's disease, Transcription, Aggregates, Inducible HD cell line, Early HD cellular phenotype, Temporal analysis, Gene clusters

## Abstract

**Background:**

Huntington’s disease (HD) is a fatal neurodegenerative disorder caused by a CAG expansion in the Huntingtin (*HTT*) gene. Proteolytic cleavage of mutant huntingtin (Htt) protein with an expanded polyglutamine (polyQ) stretch results in production of Htt fragments that aggregate and induce impaired ubiquitin proteasome, mitochondrial functioning and transcriptional dysregulation. To understand the time-resolved relationship between aggregate formation and transcriptional changes at early disease stages, we performed temporal transcriptome profiling and quantification of aggregate formation in living cells in an inducible HD cell model.

**Results:**

Rat pheochromocytoma (PC12) cells containing a stably integrated, doxycycline-inducible, eGFP-tagged N-terminal human Htt fragment with an expanded polyQ domain were used to analyse gene expression changes at different stages of mutant Htt aggregation. At earliest time points after doxycycline induction no detectable aggregates and few changes in gene expression were observed. Aggregates started to appear at intermediate time points. Aggregate formation and subsequent enlargement of aggregates coincided with a rapid increase in the number of differentially expressed (DE) genes. The increase in number of large aggregates coincided with a decrease in the number of smaller aggregates whereas the transcription profile reverted towards the profile observed before mutant Htt induction. Cluster-based analysis of the 2,176 differentially expressed genes revealed fourteen distinct clusters responding differently over time. Functional enrichment analysis of the two major gene clusters revealed that genes in the up-regulated cluster were mainly involved in metabolic (antioxidant activity and cellular ketone metabolic processes) and genes in the down-regulated cluster in developmental processes, respectively. Promoter-based analysis of the identified gene clusters resulted in identification of a transcription factor network of which several previously have been linked to HD.

**Conclusions:**

We demonstrate a time-resolved relationship between Htt aggregation and changes in the transcriptional profile. We identified two major gene clusters showing involvement of (i) mitochondrial dysfunction and (ii) developmental processes implying cellular homeostasis defects. We identified novel and known HD-linked transcription factors and show their interaction with known and predicted regulatory proteins. Our data provide a novel resource for hypothesis building on the role of transcriptional key regulators in early stages of HD and possibly other polyQ-dependent diseases.

**Electronic supplementary material:**

The online version of this article (doi:10.1186/s12864-017-3745-z) contains supplementary material, which is available to authorized users.

## Background

Huntington’s disease (HD) is a neurodegenerative disorder that manifests in behavioural changes as well as a decline in cognitive abilities and motor functions. Disease onset typically starts around the fourth decade of life and after disease onset symptoms progressively increase [1]. HD is caused by an expanded stretch of CAG repeats in the Huntingtin (*HTT*) gene which is translated into an abnormally large polyglutamine (polyQ) domain near the huntingtin (Htt) protein N-terminus. An increasing number of CAG repeats is related to progressively earlier ages of disease onset. The Htt protein is involved in a variety of cellular processes and interacts with several proteins including transcriptional regulators and nuclear receptors. Mutant Htt is known to exhibit a changed protein interaction profile and to disrupt physiological functioning of its binding partners [2–6], e.g., CREB Binding Protein (CBP) and Specificity Protein 1 (Sp1) [7–9]. Even though Htt is ubiquitously expressed in all cell types, medium-sized spiny neurons in the striatum are particularly affected very early in HD [10].

Transcriptional dysregulation is proposed to represent a key factor in HD development and major changes in gene expression are detected in HD patient post-mortem brain tissue and in disease model systems [11–18]. Protein aggregates in the brain are a hallmark of HD and transcriptional regulators are found to be associated with these aggregates [19–23]. In mouse models Htt aggregates are noted to appear before disease onset [24, 25]. Sequestration of proteins involved in transcriptional regulation into aggregates [20–23, 26] or ubiquitin proteasome dysfunction [27, 28] is suggested to represent a causative role in HD pathogenesis. However, sequestration of proteins into aggregates does not seem to affect protein concentrations to a biologically significant degree [29] and both transcriptional dysregulation and ubiquitin proteosome impairment are noted to already take place before nuclear aggregate formation. It is now thought that it is not mutant Htt aggregation but the soluble Htt protein fragments that play an important role in early cellular pathology [30–32]. Clearly, the causal relationship between protein aggregation, transcriptional dysregulation and neuronal cell dysfunction at early disease onset are not fully understood.

In the present study, we used an inducible HD cell system to determine the dynamics of early-state transcriptional changes and aggregate formation. We performed transcriptional profiling and quantified aggregate formation over time upon induction of mutant and control Htt protein expression. Our data show that a rapid accumulation of small mutant Htt aggregates coincides with a significant change in the transcriptional profile represented by one major cluster of up-regulated genes involved in metabolic processes and one major cluster of down-regulated genes involved in developmental processes. At later time points small aggregates seem to merge into larger ones and changes in gene expression diminish. We identified a number of transcription factors involved in transcriptional dysregulation and illustrate their interactions that may be part of a larger network commonly affected not only in HD, but possibly also in other polyQ-dependent disorders.

## Methods

### Cell culture

Rat PC12 cells containing exon 1 of the human *HTT* gene, with either 23 (control) or 74 (HD) CAG repeats, fused to Green Fluorescent Protein (GFP) under control of a doxycycline-inducible promoter [33] were cultured in six well plates containing high glucose Dulbecco’s modified Eagle’s medium containing pyruvate and L-glutamine (DMEM 41966, Invitrogen Life Technologies) supplemented with 100 U/ml penicillin and streptomycin (Invitrogen Life Technologies), 2 mM L-glutamine (Invitrogen Life Technologies), 10% heat-inactivated horse serum (Invitrogen Life Technologies), 5% TET-approved heat-inactivated fetal bovine serum (Clontech), 100 μg/ml G418 (Invitrogen Life Technologies) and 75 μg/ml hygromycin (Invitrogen Life Technologies) at 37 °C and 10% CO_2_. Induction of fusion protein was accomplished by incubating the cells with 1 μg/ml doxycycline (Clontech). Cells were harvested at 0 (pre-induction state), 4, 8, 24, 36, 48, 72, 96 and 120 h post-induction.

### Microscopy

Induced cells were imaged using a Zeiss Axiovert 200 M wide-field fluorescence microscope equipped with a 100x Plan-Apochromat (1.4NA) oil immersion lens and a Cairn Xenon Arc lamp with monochromator and objective heater. Images were recorded with a cooled CCD camera and analysed using a modified version of the Argos system [34].

The outline of the cells and the aggregates present in the captured images were detected as follows. A Gaussian filter with σ = 0.2 μm in combination with a threshold intensity of 30 was used to enable detection of the cells. A Gaussian difference with σ = 0.1 and σ = 0.5 μm in combination with a threshold intensity of 80 was used for spot detection. The 20% quantile value of the data was taken as the background value and subtracted from the data. For follow-up analysis, aggregate spots smaller than 1×10^−5^ μm were considered noise and discarded. For each remaining aggregate, the logarithm of its volume (log_e_(volume)) was calculated, leading to a range of values from −5.54 to 4.09. Given this range, we binned the aggregates into eleven groups. For each time point after induction, at least four images were analysed and the number of aggregates belonging to each bin was determined. To correct for the varying number of cells present in each image, the number of aggregates found for each bin was divided by the total volume of the cells found in that image.

For each of the at least four images captured per time point, an average eGFP-Htt expression value was obtained calculating the total, background-corrected fluorescence values divided by the combined cell volume.

### RNA isolation

Total RNA was extracted from PC12 cells and purified from whole cell lysates derived from samples cultured at each experimental condition. RNA isolation was performed with Trizol (Life technologies) following manufacturer’s instruction. tRNAs were removed with RNeasy MinElute clean-up kit (Qiagen). The integrity of isolated RNA was measured using the Agilent 2100 BioAnalyzer (Agilent Technologies) according to the manufacturer’s instructions. Based on the results of the integrity measurements, a quality score for each RNA sample was calculated using the RNA Integrity Number algorithm [35]. The samples used in our experiment were assigned scores ranging from 8.8 to 10, with an average score of 9.9, indicating high quality RNA samples.

### Microarray hybridisation

A total of 108 samples were prepared for hybridisation: six for each of the nine time points for both control and HD cell lines. Each test sample was labelled with Cy3 and hybridised against a Cy5-labelled common reference sample, which consisted of a pool of equal amounts of RNA from all individual test samples. Labelling of the RNA samples was performed using Ambion aminoallyl amplification. Hybridisation of the samples was randomized across nine slides containing twelve arrays each. Four samples had to be discarded because of hybridization-related problems.

The microarrays used in this study were Nimblegen-Roche chips (12–146 k format) spotted with Nimblegen’s default oligo library (corresponding to 26,419 rat genes, each of which was represented by multiple distinct probes) as well as probes to be used for internal quality controls and array construction. The Nimblegen oligo library was supplemented with probes recognizing three additional rat genes (arginine-glutamic acid dipeptide repeats (Rere), HECT, UBA and WWE domain containing 1 (Huwe1) and Specificity protein 3 (Sp3)), probes to quantify the eGFP-Htt construct expression levels and to detect reverse complementary versions of the probes targeting eGFP-Htt as negative controls. Sequences for these additional probes are included in the supplementary material (Additional file 1).

### Reannotation of microarray probes

The annotation of the probes corresponding to rat genes was updated using the Ensembl rat transcriptome (Rnor_5.0). Probe sequences were aligned to the rat transcriptome using blast with a threshold E-value of 1x10^−10^. Probes uniquely matching a transcript sequence with a bit score of at least 100 were assigned the corresponding Ensembl transcript and gene ID. Probes matching multiple transcript sequences belonging to the same gene were only assigned the Ensembl gene ID. For probes that could not be matched at all, we used Nimblegen’s original RefSeq ID annotation. Additional information such as Entrez gene IDs and gene symbols were retrieved from the ‘rnorvegicus_gene_ensembl’ dataset from the Ensembl database using the biomaRt R/Bioconductor package (version 2.18.0 [36, 37]). After reannotation, not all genes interrogated by the default oligo library were represented by four distinct probes as originally designed (Additional file 2).

### Pre-processing and statistical analysis

Microarray expression values were analysed using R statistical software (version 3.1.0) and R/Bioconductor packages. Furthermore, all microarray probe sets related to Nimblegen internal controls and array construction were removed from the dataset to avoid introducing systematic effects during pre-processing of the data. The remaining probes all corresponded to rat genes or one of the additional custom probes. The R/Bioconductor package arrayQualityMetrics [38] was used to assess whether the microarray data were of good quality. No outlier arrays were observed.

Expression values were background corrected using the normal-exponential (normexp) method (limma package, version 3.20.1 [39]), with an offset value of 20 to shrink log-transformed red/green ratios towards zero at lower intensities. Loess normalisation (limma package) of the log-ratios was performed to mitigate dye biases within each array. The expression values of all probes corresponding to the same Ensembl gene ID were summarized to a single representative expression value through the median polish procedure (affy package, version 1.42.2 [40]). When lacking an updated Ensembl gene ID, the original RefSeq gene IDs were used to summarise the probes. Significance of differential expression was assessed using linear modelling and empirical Bayes moderated statistics (limma package). The gene-wise linear models employed included a factor for the combination of cell line (control/HD) and time point, and a factor to correct for potential slide effects present in the data. For both control and HD samples, contrasts were made between each individual time point t and the pre-induction state for that cell line, that is, C_t_-C_0_ and HD_t_-HD_0_ respectively. We also investigated which genes responded differently over time in the HD cell line relative to the control cell line to obtain a HD-specific set of significant differentially expressed (DE) genes (HD_t_-HD_0_) - (C_t_-C_0_). Correction for multiple testing was done using the Benjamini-Hochberg false discovery rate.

### Cluster and enrichment analyses

Expression values were corrected for potential slide effects using ComBat (sva package, version 3.4.0 [41]) with ‘slide’ as a batch covariate and a model matrix containing factors for the cell lines and time points used. Only genes annotated with an updated Ensembl gene ID and a single Entrez gene ID were used. Expression values of the 2176 HD-specific genes were used for further analyses using Expander (version 6.0) [42]. Expression values derived from arrays representing the same conditions were averaged to a single expression value. Next, genes were clustered by expression pattern using the CLICK algorithm implemented in Expander [43] with the ‘Default Homogeneity’ option and using Pearson correlation as a distance measure.

The Entrez IDs for the genes comprising the resulting clusters were then used for functional enrichment analysis using the TANGO algorithm with default settings. Reported *p*-values are the result of testing for the significance of overlap between gene groups and Gene Ontology (GO) terms using a hypergeometric test. P-values were corrected for multiple testing by sampling for each cluster 2,500 random gene sets of the same size as the original cluster and estimating the empirical p-value distribution for the evaluated GO terms. All rat genes represented on the array by probes for which we had updated annotation (Additional file 2) were used as background set. GO terms with a corrected *p*-value smaller than 0.05 were considered to be significant.

For each of the clusters created by CLICK, promoter analysis was carried out to identify transcription factors for which binding sites were overrepresented in the promoter regions of a set of genes. The PRIMA algorithm [44] was used to analyse a region from 1000 bases upstream to 200 bases downstream of the transcription start site of each gene. All genes represented on the array for which we had updated annotation were used as background set. Binding site motifs with a *p*-value < 0.0001 as determined using a generalized hypergeometric test were considered significant.

### Protein interaction analysis

Interaction between proteins was studied using the Search Tool for the Retrieval of Interacting Genes/Proteins (STRING) and its database of known protein interactions (http://string-db.org/; version 10.0). Gene symbols were used as input while selecting Homo sapiens as species. The network was created based on high confidence interactions (cut-off score of 0.7) and visualised using the ‘Molecular Action’ view option to show the type of interaction between the nodes.

## Results

### Onset and accumulation of mutant HD aggregates

**Fig. 1:**
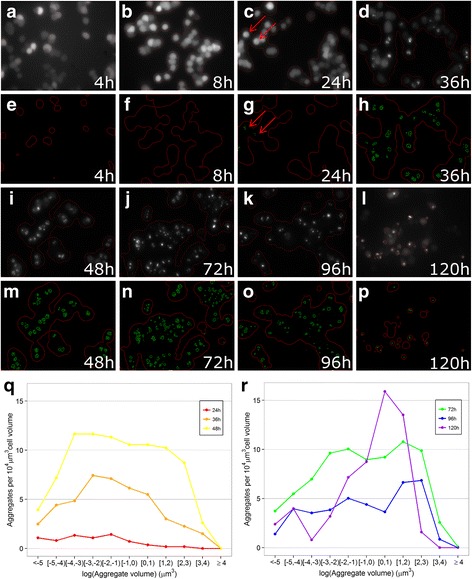
eGFP-Htt-Q74 aggregate formation upon doxycycline induction. PC12 cells expressing eGFP-Htt-Q74 HD upon doxycycline induction were imaged at various time points post-induction (4 – 120 h) using fluorescence living cell imaging. Image analysis was performed using a modified version of the Argos system, an interactive setup to combine numerical analysis with visual analysis (**a**-**p**, representative examples of at least four replicates per time point). **a**-**d** and **i**-**l** show the Htt-Q74 eGFP expressing cells at the various post-induction time points. Cell outlines (*red*) at different time points were detected by image analysis. **e**-**h** and **m**-**p** show the intracellular eGFP-Htt-Q74 aggregates (*green*) detected in each image at the different time points. *Red arrows* in **c** and **g** point to the aggregates at time point 24 h post-induction. Aggregate formation was first observed in cells around 24 h post-induction. Cell and aggregate volumes were calculated in at least four images at each time point (24–120 h after doxycycline induction). Aggregates detected in cells were binned based on their log_e_(volume). The number of aggregates per cell volume was determined for each bin and plotted separately for the intermediate (24-48 h, panel **q**) and late (72-120 h, panel **r**) time points

We used fluorescence microscopy to study the temporal expression of eGFP-tagged N-terminal Htt fragments with an expanded polyQ domain in PC12 cells (Q23 as control and Q74 as HD cell line). We determined the localization and aggregation of eGFP-Htt-Q74 at 4 h - 120 h after doxycycline induction (Fig. 1a-p). Additional file 3 shows the eGFP-Htt-Q23 expressing cells at 4 h - 120 h after doxycycline induction. We did not detect aggregate formation in the Q23 control cell line at any of the post-induction time points. We used Argos software to calculate the volume of each individual aggregate and the combined volume of all cells per image (Fig. 1e-h, m-p).

Aggregates detected in cells were binned based on their log_e_(volume). The first eGFP-Htt-Q74 aggregates were observed 24 h post-induction and were relatively small, mainly occurring in the first 6 bins (Fig. 1q). Over time, the number of aggregates increased until it reached a maximum at 48–72 h post-induction. In this same time frame, the distribution of the aggregate sizes started shifting towards larger volumes, occurring in almost all bins (Fig. 1r). At 96 h post-induction, the total number of aggregates decreased notably. Surprisingly, at 120 h post-induction in a defined set of aggregate units (bins [0,1) and [1,2)) a large increase in the number of aggregates per cell volume was noted compared to the number of aggregates per cell volume observed at 48–72 h post-induction.

### Doxycycline-induced expression of eGFP-Htt

**Fig. 2:**
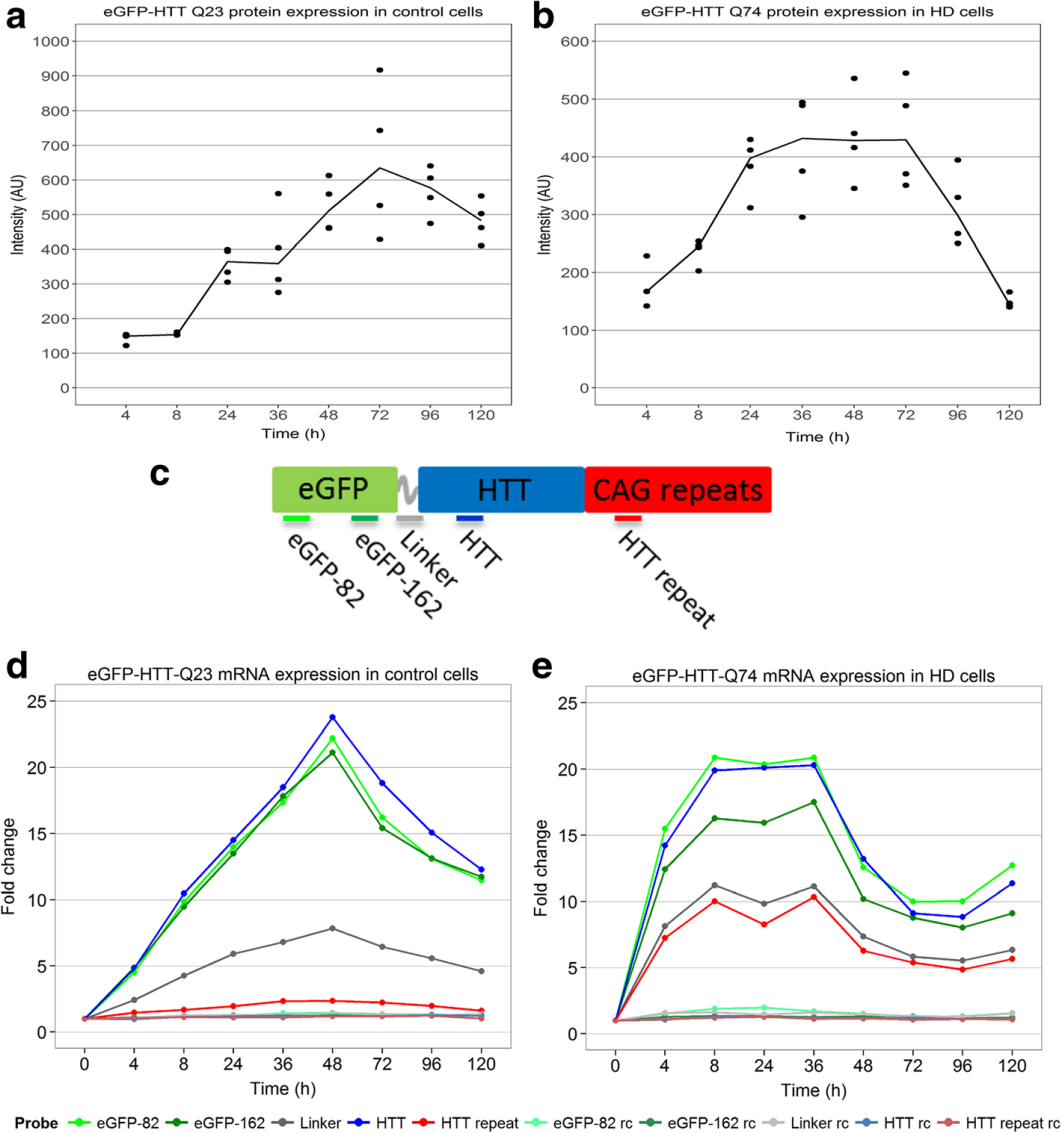
Expression of the eGFP-Htt construct. Induction of the eGFP-Htt construct was quantified by fluorescence measurements in the microscopy images and through the use of a custom probe set spotted on the arrays. Expression of eGFP-Htt protein was obtained by calculating total measured fluorescence divided by the combined cell volume for each of the at least four images analysed per time point for control (**a**) and HD (**b**) cells. The solid line connects the median values of each time point. **c** The hybridisation sites for the microarray probes designed to detect the eGFP-Htt mRNA are indicated. For each eGFP-Htt probe, the fold change in expression level over the pre-induction state (0 h) was calculated and plotted for each sampled time point in control (**d**) and HD (**e**) cells. Probes with the reverse complementary (rc) sequence of the expression probes were used as negative controls

The eGFP-Htt protein expression levels were estimated based on fluorescence measurements using the images described earlier (Fig. 2a, b). Upon doxycycline induction, we observed a swift eGFP-Htt-Q74 protein induction for up to 24 h at which point the protein reached a maximum expression level. The eGFP-Htt-Q74 expression level remained unchanged until 72 h and then subsequently decreased, reaching levels at 120 h post-induction similar to those observed after 4 h. eGFP-Htt-Q23 protein levels are increasing more gradually and reach a slightly higher plateau level compared with eGFP-Htt-Q74 levels.

To determine the changes in gene expression in the eGFP-Htt-Q23 control and eGFP-Htt-Q74 HD cell lines, mRNA was harvested in parallel to the microscopic aggregate formation analysis, including the pre-induction state. We used the Nimblegen-Roche 12-146 k microarray platform to analyse temporal gene expression at defined post-induction time points. Several probes were included to measure eGFP-Htt expression levels (Fig. 2c). Additional File 4 shows for each condition the mean log2-ratio with respect to the Cy5-labelled common reference sample of these probes as well as of the probe targeting endogenous rat *Htt* (ENSRNOG00000011073). Our data show that levels of the probe targeting endogenous *Htt* did not change upon doxycycline induction in the eGFP-Htt-Q23 control and eGFP-Htt-Q74 HD cell line.

The expression values of these probes were pre-processed along with all other probes on the microarrays. Based on these processed expression values the fold changes in eGFP-Htt expression with respect to the pre-induction state were calculated for each time point in both the control and the HD cells (Fig. 2d and e, respectively). Expression of eGFP-Htt mRNA increased rapidly after doxycycline induction. In the HD cells the doxycycline-induced expression increased faster than in the control cells. Similar to the observed eGFP-Htt-Q74 protein levels, mRNA expression in HD cells showed a peak at the early post-induction time points (4–8 h), followed by a fast decrease (36 h) and a subsequent drop to end-point levels (>48 h). In control cells, we observed a gradual increase, with eGFP-Htt mRNA expression levels reaching a peak at 48 h post-induction, followed by a decrease in expression. Probes with a reverse complementary (rc) sequence to the eGFP-Htt probes were used as negative controls. None of the rc probes exhibited a change in expression level as compared to the pre-induction state during the course of the experiment.

In both control and HD cells, a similar trend was observed for the eGFP-82, eGFP-162, linker and Htt probes (Fig. 2d, e). The only clear difference in comparing control and HD cells is noted in the expression of the probe recognizing the Htt repeat region of the construct. Here, we did not observe an increase in expression in control cells, whereas in the HD cells the expression pattern was similar to that observed for the linker probe. The increase in CAG repeat sequences in the eGFP-Htt-Q74 compared to the -Q23 control may explain improved hybridisation of this probe to the Htt-Q74 site.

### Doxycycline induced changes in gene expression profiles

**Fig. 3:**
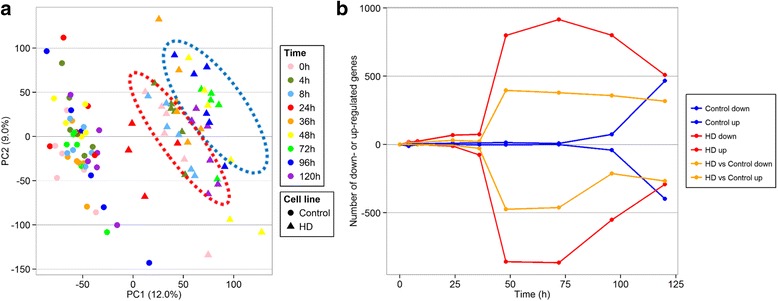
Changes in gene expression in the Htt-Q23 control versus Htt-Q74 HD cell line. **a** First two principal components of the pre-processed microarray data corrected for potential slide effects using ComBat. Each symbol corresponds to a single microarray. The different time points are shown in colour. Dots represent the data obtained from the Htt-Q23 control cell line and triangles the data from the Htt-Q74 HD cell line. The first principal component (PC1) separates control from HD microarrays whereas the combination of PC1 and PC2 separates early (0-36 h, indicated in the red oval) from later (48-120 h, indicated in the blue oval) time points in the Htt-Q74 HD cells. Three of the 24 h samples from the HD cell line did not group with the other early samples, however. This separation between early and late samples was not observed in the Htt-Q23 control cells. The numbers on the axes indicate the percentage of variance explained by the plotted principal components. **b** Changes in the number of significantly DE genes were examined over time. Expression values of all genes at each time point were compared to pre-induction state (time = 0 h). Positive numbers on the y-axis indicate the number of genes found to be up-regulated whereas negative numbers indicate the number of down-regulated genes (adjusted *P*-value < 0.05). The change in the number of DE genes over time is plotted separately for the control (*blue*) and HD (*red*) samples and finally for the HD samples after correction for the response detected in the control samples (*orange*)

Using a Nimblegen-Roche 12-146 k microarray platform, we assayed approximately 26 k rat genes for transcriptional changes at regular intervals over a period of 120 h after induction of eGFP-Htt-Q23 (control) or eGFP-Htt-Q74 (HD). Principal Component Analysis (PCA) of the processed microarray data revealed a clear separation between the majority of control and HD samples based on the first principal component (Fig. 3a). Note that the HD samples taken 24 h post-induction did not group well with the other HD samples. Furthermore, a combination of the first and second principal component separated the HD samples into an ‘early’ (0-8 h, red oval) and ‘late’ (36-120 h, blue oval) cluster. A similar time-based clustering of the control samples was not as evident, indicating that the changes between control samples over time were smaller than the changes between HD samples.

We compared the expression values of genes in the control and HD cells before doxycycline induction to assess the baseline differences between the two cell lines (Additional file 5). The control and HD cells express the eGFP tagged *HTT* gene under control of a doxycycline promoter in a distinct genomic site. A set of 2059 genes were significantly differentially expressed (DE) (adjusted *P*-value < 0.05) between control and HD cells. Functional enrichment analysis on this set of genes revealed an overrepresentation of Gene Ontology (GO) terms (representing biological processes or molecular functions) predominantly related to cell division, apoptosis and stress responses (data not shown). Closer examination revealed that all five eGFP-Htt probes are included in this set of baseline DE genes and exhibited increased expression levels in the HD cells compared to the control cells. These findings indicate a clear and substantial difference between the two cell lines even before induction of the eGFP-Htt constructs. The baseline expression levels in the eGFP tagged polyQ HTT gene in the control and HD cells depend on the genomic integration site of the gene since doxycycline-induced tet-on gene expression systems exhibit relatively strong promoter induction. To prevent genes from being reported as DE due to transcriptional differences present in control versus HD samples before doxycycline induction, we compared measurements between each time point and the pre-induction state for both cell lines separately. We also determined which genes responded differently over time in the HD cell line compared to the control cell line, referred to as HD-specific genes. The latter analysis led to a set of 2,176 DE genes that were found to be DE (moderated F-statistics, adjusted *P*-value < 0.1) in at least one time point compared to the pre-induction state (Additional file 6).

Overall very few genes are significantly DE in the control cells prior to 120 h post-induction (Fig. 3b). In the HD cells we detected only few changes in gene expression before 36 h post-induction, especially after correcting for effects in the control cell line (Fig. 3b, orange lines). Roughly half the genes that were DE in the HD samples before 36 h post-induction dropped below the significance threshold after correcting for changes observed in the control samples. This suggests that in this time frame these genes exhibit a similar but non-statistically significant up- or down-regulation in the control cell line. These genes could exhibit an altered expression either due to doxycycline-induced overexpression of the *HTT* gene or due to a mutant Htt protein-induced gain-of-toxic function. At 36 h post-induction, the number of DE genes in HD samples greatly increased, both before and after correcting for changes observed in the control samples. Upon 72 h post-induction, the number of DE genes in the control samples started to increase, slowly at first but much more rapidly after 96 h. We found roughly equal amounts of up- and down-regulated genes in the HD and control samples. Both control and HD cells exhibited around 800 DE genes at 120 h post-induction and 141 of these genes were found to be DE in both control and HD cell lines at this time point. Out of this set, 55 genes exhibited increased expression in control cells while showing decreased expression in the HD cells. The remaining genes were all up-regulated in both cell types. For each time point, roughly half of the DE genes were also DE in the same direction for adjacent time points, indicating a more persistent change in expression.

**Fig. 4:**
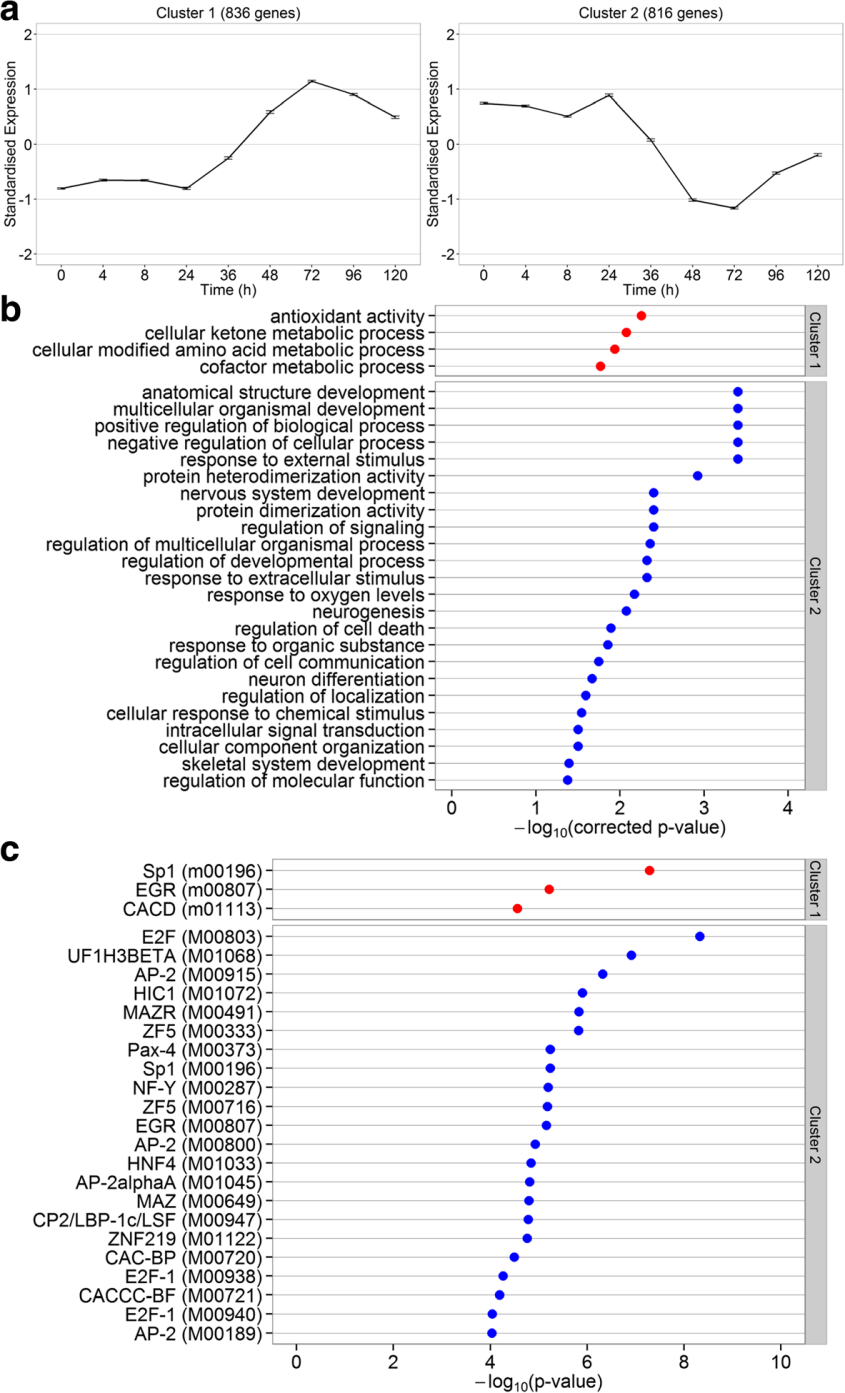
Expression profile clustering and enrichment analyses. **a** DE genes were clustered based on their expression profiles in HD cells. The two largest clusters created by the CLICK algorithm show a gradual increase and decrease in gene expression, respectively. Expression patterns were standardized to mean zero and standard deviation one. Error bars correspond to one standard deviation. **b** The gene clusters were subjected to a functional enrichment analysis using the TANGO algorithm to identify overrepresented biological processes and/or molecular functions. The –log_10_(corrected *P*-values) of all functional terms found to be significant are plotted. Terms found to be overrepresented in cluster 1 are indicated in *red*, those found in cluster 2 are indicated in *blue*. When correcting for multiple testing, no overrepresented functional terms could be detected for any of the twelve other clusters. **c** A transcription factor binding motif enrichment analysis of the promoter regions of the clustered genes was performed using the PRIMA algorithm. The –log_10_(*P*-values) of all motifs found to be significant were plotted. Motifs found to be overrepresented in cluster 1 are indicated in *red*, those found in cluster 2 are indicated in *blue*. For each transcription factor the corresponding TransFac DB accession number is listed between parentheses on the y-axis

We performed a cluster-based analysis to study the temporal behaviour of the HD-specific set of genes (Additional file 6). Fourteen distinct clusters were found that represented all of the 2176 selected genes (Fig. 4a and Additional file 7). The large majority of genes showed either one of two distinct patterns illustrating either an increased (Fig. 4a; cluster 1) or decreased expression (Fig. 4a; cluster 2) from around 24 h post-induction. Regardless of the direction (up or down) of the change in expression, a maximum effect was reached around 72 h. In general, few genes exhibited changes in expression before 24 h post-induction.

### Functional enrichment analysis of gene clusters

We subjected the obtained gene clusters to functional enrichment analysis to identify overrepresented GO terms. Significantly overrepresented biological processes and molecular functions were only detected for gene clusters 1 and 2 (Fig. 4b). Cluster 1 was enriched for genes involved in metabolic processes, whereas the genes in cluster 2 were mostly involved in developmental processes. The two most significantly overrepresented GO terms in cluster 1 represent ‘antioxidant activity’ and ‘cellular ketone metabolic processes’.

To identify potentially pathogenic processes during early HD, we investigated the transcriptional changes at time points before 36 h post-induction in more detail. Given the modest changes in expression for these early time points, we selected HD-specific DE genes at the 4, 8 and 24 h time points using a less strict significance threshold (nominal *P*-value < 0.2). To select the more persistent changes in gene expression during this time frame, the resulting set was limited to those genes that were found DE at all three time points. A cluster analysis and subsequent functional enrichment analysis on this set of 404 ‘early response’ genes revealed only processes related to cell division and DNA replication to be overrepresented (Additional file 8). These processes were all detected in a single cluster comprised of genes being persistently down-regulated until the 72 h time point with exception of the 36 h time point (Additional file 8, panel A).

### Promoter enrichment analysis of gene clusters

Promoter-based enrichment analysis of the observed gene clusters resulted in the identification of a number of transcription factors with binding sites overrepresented in the promoter regions of the genes included in each cluster (Fig. 4c). Among these transcription factors we observed several transcription factors that have previously been linked with HD such as Sp1, Nuclear transcription factor Y (NF-Y), E2F and Hypermethylated in cancer 1 (HIC1) [7, 8, 45–47].

We used the STRING database of known and predicted protein interactions to find potential relationships between the transcription factors identified in the promoter analysis of our detected DE genes. The transcription factors identified were used as input for STRING. The selected proteins were connected through known and predicted interactions and a small network could be established (Additional file 9).

## Discussion

HD is a monogenic neurodegenerative disease displaying widespread transcriptional deregulation as a key feature resulting from a CAG expansion in the *HTT* gene on chromosome 4p16.3 leading to the production of mutant Htt protein containing a prolonged polyQ tract that is prone to aggregation. The CAG repeat length is the major determinant of the age of onset although significant variability in the age of onset exists, underscoring disease complexity. There is an unmet need for disease-modifying therapies as currently available treatments can only ease disease symptoms. Knowledge of the cause(s) of early onset events provides important information to infer disease state-specific signatures to be used for diagnostic screening and to find targets for disease state-specific interference. Transcriptional deregulation and aggregate formation are key events of early disease onset but their temporal relationship during the earliest cellular changes is largely unresolved.

In the present study, we determined the time-resolved relationship between aggregate formation and changes at transcriptional level during early disease stages in an inducible HD cell model. Obviously, HD is a disease mostly known to occur in neuronal cells and in vitro cell line models have clear limitations to recapitulate HD in vivo pathology. However, considering the late onset of HD disease pathology and the large variability in disease pathology outcome, both in vitro and in vivo experimental models of HD have been invaluable to interpret disease pathogenesis, especially in the early phase of the disease. It is important to realize the challenges of using cell models, i.e., our analysis is based on control and HD cell lines that are intrinsically different, which clearly hampers analysis to interpret differentially expressed genes. It should however be noted that when analyzing patients intrinsic differences between patients are even worse and thereby largely complicating mechanistic insight in early onset phenomena. In this study, we used rat pheochromocytoma cells (PC12) containing a stably integrated doxycycline-inducible eGFP-tagged N-terminal Htt fragment with an expanded polyQ domain as a cell model to study early-state changes in Huntington’s disease. These cells have been well characterized and used by the field. The behavior of cells expressing the eGFP tagged Htt fragment has been extensively studied [48]. Our data show that the kinetics of exon1 mRNA and protein levels in particular of differentially expressed genes in control and HD cell line are different. The eGFP-Htt-Q23 control and eGFP-Htt-Q74 HD cell lines have different baseline expression levels. Therefore, we did not compare individual time points between HD and control samples, but determined which genes responded differently over time between the two cell lines, thus taking differences at baseline into account thereby also correcting for the potential effect of adding doxycylin to the cells.

Our data indicate that the earliest cellular changes can be divided into three phases with distinct cellular phenotypes. The first alteration in transcriptional activity (before the 24 h post-induction time point) occurs in the absence of microscopically detectable aggregates. We observed that in this first phase deregulated genes are typically involved in cell division and DNA replication suggesting that reduced cellular replicative ability might represent a very early effect of Htt polyQ induction. It is intriguing that we detect deregulation of genes involved in cell division and DNA replication in our cell model. Possibly deregulation in these genes reflects a general hampered cell functioning causing overall cellular deregulation of main survival genes in an early phase. However, there is growing evidence that also glial cells are affected in HD and play a major role in the disease pathology [49, 50], making our findings on deregulated genes involved in cell division and replication interesting. Most likely genes affected prior to 24 h post-induction may be particularly sensitive to disruption of their normal functioning by the presence of polyQ-expanded Htt. The second phase (24–36 h post-induction) exhibits significant transcriptional deregulation coinciding with the appearance of the first small aggregates. At later time points when the number of small aggregates drops and large aggregates simultaneously increase (possibly illustrating small aggregate merging) a decrease in transcriptional deregulation is noted that might represent a protective effect due to the sequestration of polyQ-expanded Htt protein into these large aggregates.

Our aggregate volume measurements confirm previous observations in the same cell line [51], although contrary to Gong et al. (2008), we observe normal growth in the total cell volume (indicating healthy cellular viability) up to 72 h post-induction. Only images captured post-96 h show a substantial decrease in total cell volume (data not shown). Intermediate polyQ-expanded Htt accumulations have been shown to occur before the formation of full sized aggregates [52]. Arrasate et al. (2004) illustrated that neuronal survival was negatively impacted by the concentration of diffuse polyQ-expanded Htt. The presence of aggregates was shown to reduce the concentration of diffuse polyQ-expanded Htt and an increased neuronal survival [53]. It has been suggested that monomeric and oligomeric polyQ-expanded Htt protein exhibit cellular toxicity while formation of larger aggregates provides (at least temporary) protection from these toxic effects [52, 54]. These larger aggregates might eventually exhibit secondary pathogenic effects such as physically blocking vesicle or mitochondrial trafficking in the cell.

To understand which biological pathways are associated with the early-state temporal transcription changes, we used a clustering algorithm to group genes with similar expression patterns followed by a functional enrichment analysis. We identified a number of biological processes overrepresented in up- or down-regulated clustered genes that occur at the intermediate onset. The gene cluster representing upregulated genes shows overrepresentation of genes related to antioxidant activity and ketone metabolism whereas the down-regulated cluster of genes exhibits overrepresentation of several processes involved in development.

Our findings are in line with a study by van Roon-Mom et al. that analysed DE genes at day 1 and 5 after induction using the same cell line [55]. Van Roon-Mom et al. identified mitochondrial (dys)function, oxidative stress responses and a set of genes involved in dopamine biosynthesis as early cellular onset phenomena. We compared our findings with this previous study. To this end, we investigated the transcriptional profiles of the gene set identified by van Roon-Mom et al. consisting of 21 Nrf2-regulated genes in our microarray data. We observed that eight of these genes were also found to be significantly DE in our HD-specific gene set and that these genes showed the same direction of change (up or down) as found by van Roon-Mom et al. (Additional file 6). Eleven of these genes were not significantly DE in our dataset. However, most of these genes did show a change in expression level in the same direction as was found by van Roon-Mom et al. The remaining two genes were not represented by probes on our array and could therefore not be examined. Furthermore, all five genes involved in dopamine biosynthesis identified by van Roon-Mom et al. were also found to be significantly down-regulated in our study (Additional file 6). In addition to the van Roon-Mom et al. study we show the trend of DE genes at a number of intermediate time points. Especially after 3 days we observe more extensive transcriptional deregulation. Taken together our study is a follow-up analysis of the previous transcription analysis study in the PC12 HD cell model.

Mitochondrial dysfunctionality and impaired energy (glucose) metabolism are well-known phenomena associated with Huntington’s disease, as well as other neurodegenerative disorders such as Parkinson’s disease or Amyotrophic Lateral Sclerosis (ALS) [56–59]. If neurons are unable to generate sufficient energy from glucose due to a limited glucose availability or ability to metabolise glucose, neurons may increase the production of enzymes capable of utilising an alternative energy source. At impaired glucose levels, ketone bodies are an alternative source of energy for neurons [60]. Intriguingly, the overrepresentation of developmental processes suggests that HD belongs to the category of developmental disorders, which also includes schizophrenia, familial Alzheimer’s disease and Spinocerebellar ataxia 1 (SCA-1) [61]. Studies of pre-symptomatic individuals carrying an expanded *HTT* gene have shown subtle developmental defects [62]. Affected patients often experience weight loss besides severe motor abnormalities [63, 64]. In addition, pre-symptomatic children carrying the Htt polyQ expansion showed a lower body mass index [65]. Moreover, brain scans reveal that HD patients have an enlarged cortex size and that pre-symptomatic individuals have a decreased intracranial volume [66]. Energy regulation, cellular homeostasis and brain growth could typically represent early HD symptoms that arise before well-known HD symptoms and that predispose neurons to die later in life [64].

We identified a number of transcription factor binding sites that were overrepresented in promoter regions of the observed DE genes. Some of these transcription factors have previously been directly or indirectly linked to HD, i.e., Sp1, NF-Y, E2F, HIC1 and EGR-1 (Fig. 4c). It should be noted that in addition to promoter enrichment, indirect effects of regulatory or coding or non-coding regions could also be involved in causing an altered gene expression profile. Our data show that binding sites for EGR(−1) and Sp1 are overrepresented in both the up- and down-regulated genes in the early-onset gene clusters. EGR-1 and SP1 might exhibit a competitive interplay in genes that contain overlapping binding sites in their promoters for these transcription factors [67]. Interaction between Sp1 and mutant Htt has been suggested to mediate neurotoxicity but on the other hand decreased Sp1 activity has been shown to be beneficial for HD mice [68]. Deregulation of Sp1 activity through alteration of the Sp1 transcriptional complex may be responsible for most of the deleterious effects observed for Sp1, which may be ameliorated by an overall reduction of Sp1 activity. EGR-1 plays a role in the development of striatal medium spiny neurons [69], the cell type most affected by HD, in which its expression is shown to be regulated by the D1 and D2 dopamine receptors [70]. Reduced expression of D1 and D2 dopamine receptors has been detected in post-mortem tissues in HD patients [71]. We did not detect differential expression of dopamine receptors or EGR-1 (Additional file 6). The differences observed in EGR-1 activity are likely to result from more down-stream transcriptional deregulation.

We identified several particularly interesting transcription factors (Fig. 4c) that might provide a link between HD and other degenerative disorders caused by polyQ-expanded proteins, such as SCA17 and Spinobulbar Muscular Atrophy (SBMA). In SCA17, the activity of trimeric transcription factor NF-Y was shown to be reduced through sequestration of component A (NF-YA) by polyQ-expanded TATA-box binding protein (TBP) [72]. In HD cell systems and the brains of HD mice, two of the NF-Y subunits were found sequestered in mutant Htt aggregates. Moreover, binding of NF-Y to the Heat Shock Protein 70 (Hsp70) promoter was noted to be decreased in HD brain resulting in a decreased heat-shock-induced chaperone response [47]. We noted that NF-Y associates with down-regulated genes but we did not detect a significant change in the expression levels of heat-shock proteins. Possibly, down-regulation of these genes requires additional regulatory elements or signalling that occurs in the fully-differentiated neurons present in brain tissue or neighbouring cells of a different phenotype. The MYC-associated Zinc finger protein (MAZ) and related factor (MAZR) have both been implicated in regulating the expression of the androgen receptor (AR). Intriguingly, polyQ-expansion of AR leads to Spinal-Bulbar Muscular Atrophy [73, 74]. Two other transcription factors, CP2 and Zinc Finger protein 219 (ZNF219), have been implicated in the development of Alzheimer’s [75] and Parkinson’s disease [76], respectively. Alzheimer’s and Parkinson’s disease are not caused by repeat expansions in a single characteristic gene although these neurodegenerative disorders exhibit an advanced age disease onset similar as noted in HD. The transcription factors CP2 and ZNF219 might represent a change in transcriptional regulation that is generally noted in neurological diseases. A recent study in which Narayanan et al. analysed differential co-expression between gene pairs in expression data from patients suffering from Alzheimer’s or Huntington’s disease and non-diseased healthy controls showed a pattern of shared deregulation of gene expression between both sets of patients [77].

## Conclusion

Our data support the current theory that small mutant Htt aggregates are more toxic to the cell than larger ones, at least regarding transcriptional dysregulation. The DE genes that we identified are enriched for antioxidant activity and energy metabolism, underscoring the importance of mitochondrial dysfunction and impaired developmental processes in early disease progression. We identified novel HD transcription factors and regulators using our time-resolved gene expression analysis. We also confirmed a number of transcription factors that previously have been linked to HD. We constructed a network of identified transcription factors that may play an important role in the early cellular phenotype and that is likely part of a larger network typically affected in neurodegenerative diseases [9, 21, 22, 78]. Identification of key regulators in our determined network may reveal avenues for active modulation of gene expression in Huntington’s disease and other polyQ-dependent disorders.

## Additional files

Additional file 1: Additional probes used on the microarray. Probes added to the default Nimblegen library to interrogate additional rat genes (A) or as expression controls for eGFP-Htt levels (B). (PDF 34 kb)

Additional file 2: Rat gene representation by microarray library probes. Distribution of the number of probes that represent the various rat genes interrogated by the Nimblegen default probe library after reannotation. The number of genes targeted by probes that could be associated with an Ensembl gene ID are indicated in the second column. The third column indicates the number of genes represented solely by probes that could not be linked to an Ensembl ID. In these cases the original RefSeq IDs were kept. (XLSX 10 kb)

Additional file 3: Absence of eGFP-Htt-Q23 aggregate formation upon doxycycline induction. PC12 cells expressing eGFP-Htt-Q23 (control) upon doxycycline induction were imaged at various time points post-induction (4 – 120 h) using fluorescence living cell imaging. A-H show the Htt-Q23 eGFP expressing cells at the various post-induction time points (representative examples of at least four replicates per time point). (PPTX 647 kb)

Additional file 4: The table shows the mean log2-ratio with respect to the Cy5-labelled common reference of all probes used to measure eGFP-Htt expression levels, i.e. eGFP-82, eGFP-162, Linker, HTT, HTT repeat, eGFP-82 rc, eGFP-162 rc, Linker rc, HTT rc, HTT repeat rc (Fig. 2c; rc: reverse complementary) and also the probe targeting endogenous *Htt* (ENSRNOG00000011073) upon doxycline induction in the Q23 control and Q74 Htt cell line. The expression values of these probes were pre-processed along with all other probes on the microarrays and the fold changes in eGFP-Htt expression with respect to the pre-induction state (0 h) in control and HD cells were calculated for each time point as shown in Fig. 2d, e. (XLSX 15 kb)

Additional file 5: Expression values of genes (with Ensembl Gene ID) in the control and HD cells were compared before doxycycline induction to assess the baseline differences between the two cell lines. A set of 2,059 genes were significantly differentially expressed (adjusted *P*-value < 0.05) in control and HD cells. (XLSX 182 kb)

Additional file 6: Huntington-specific differentially expressed genes. Genes (with Ensembl Gene ID) found to be DE in at least one time point (adjusted *P*-value < 0.1, moderated F-test) compared to the pre-induction state in the HD samples after correction for effects observed in the control samples. Genes are identified by Ensembl and Entrez Gene ID and gene symbol in the first three columns. The (adjusted) overall *P*-value gives the (adjusted) P-value for a gene to be significantly DE in at least one time point measured in the experiment. Each gene was placed in a cluster as determined by the CLICK algorithm. FC shows the fold change in expression compared to the pre-induction state with (adjusted) *P*-value listing the (adjusted) P-value resulting from significance testing of differential expression of the gene at that time point. Nrf2-regulated genes and genes involved in dopamine biosynthesis identified by van Roon-Mom et al. [55] are highlighted in yellow and blue, respectively. (XLSX 891 kb)

Additional file 7: Expression profiles for all fourteen clusters created using the CLICK algorithm (Expander). Expression patterns for all clusters were standardized to mean zero and standard deviation one. Error bars correspond to one standard deviation. (PDF 360 kb)

Additional file 8: Expression profile and functional enrichment analysis of early persistently DE genes. (A) Genes were selected from the HD-specific set of DE genes and further limited to those exhibiting a significant change in expression level during the full first 24 h post-induction using a relaxed significance threshold (*P*-value < 0.2). The resulting set of 404 genes was then subjected to cluster analysis followed by functional enrichment analysis. (B) Only one cluster was found to contain genes that overrepresented biological processes. Error bars correspond to one standard deviation. (PDF 224 kb)

Additional file 9: Network of interactions between the transcription factors identified in the promoter analysis of DE genes in our experiment. Interactions between the transcription factors identified in the PRIMA analysis were extracted using the Search Tool for the Retrieval of Interacting Genes/Proteins (STRING) and its database of known protein interactions (http://string-db.org/; version 10.0). Gene symbols were used as input while selecting Homo sapiens as species. The network was created based on high confidence interactions (cut-off score of 0.7) and visualised using the ‘Molecular Action’ view option to show the type of interaction between the nodes. The table shows the name of the retrieved transcription factors, the alias used in STRING and a few comments. The network illustrates the detected connections. (PDF 521 kb)

## Abbreviations

ALS: Amyotropic lateral sclerosis
AR: Androgen receptor
CBP: CREB binding protein
DE: Differentially expressed
EGR-1: Early growth response protein 1
GO: Gene ontology
HD: Huntington’s disease
HIC1: Hypermethylated in cancer 1
Hsp70: Heat shock protein 70
Htt: Huntingtin
HUWE: HECT, UBA and WWE domain-containing protein 1
MAZ: Myc-associated zinc finger protein
MAZR: MAZ-related factor
NF-Y: Nuclear factor Y
NI: Nuclear inclusion
Nrf2: NF-E2 related factor 2
PCA: Principal component analysis
PolyQ: Polyglutamine
rc: reverse complementary
RERE: Arginine-glutamic acid dipeptide repeats
SBMA: Spinobulbar muscular atrophy
SCA-1: Spinocerebellar ataxia-1
Sp1: Specificity protein 1
TBP: TATA-box binding protein
TGFβ: Transforming growth factor β
ZNF219: Zinc finger protein 219
