# Supplementary material for: The dynamics of early-state transcriptional changes and aggregate formation in a Huntington’s disease cell model
Source: BMC Genomics. 2017 May 12;18:373. doi: 10.1186/s12864-017-3745-z (PMC5429582; doi:10.1186/s12864-017-3745-z)
Supplement: Supplementary file 1 — Additional probes used on the microarray. Probes added to the default Nimblegen library to interrogate additional rat genes (A) or as expression controls for eGFP-Htt levels (B). (PDF 34 kb) [file 12864_2017_3745_MOESM1_ESM.pdf]

Table A: Custom rat gene probes added to microarray library

	Gene Symbol	Ensembl ID
1.	Rere	ENSRNOG00000017940
	5’-GAGATAACGTCTTACATCACTGAGGATGATGTTGTTTACAGACCAGGAGGTAAGACTCAG-3’	
2.	Sp3	ENSRNOG00000019263
	5’-AGTTGCCAAATCTACAGACAGTTACAGTAAATTCTATAGATTCTACTGGTATACAGCTAC-3’	
3.	Huwe1	ENSRNOG00000002877
	5’-CTACTTTACTAGGTCTTTCTACAAACACATCTTGGGCAAATCTGTCAGGTATACAGATAT-3’	

Table B: Custom construct expression control probes added to microarray library

	Probe name	Orientation
1.	eGFP-82	Normal
	5’-CGTAAACGGCCACAAGTTCAGCGTGTCCGGCGAGGGCGAGGGCGATGCCACCTACGGCAA-3’	
2.	eGFP-162	Normal
	5’-ACTACCTGAGCACCCAGTCCGCCCTGAGCAAAGACCCCAACGAGAAGCGCGATCACATGG-3’	
3.	Linker	Normal
	5’-CCGCCTCCTCAGCTTCCTCAGCCGCCGGATCTATGAAGGCCTTCGAGTCCCTCAAGTCCT-3’	
4.	HTT	Normal
	5’-GATCTATGAAGGCCTTCGAGTCCCTCAAGTCCTTCTTTTTCTCAGCTTCCTCAGCCGCCG-3’	
5.	HTT repeat	Normal
	5’-CAGCAGCAGCAGCAGCAGCAGCAGCAGCAGCAGCAGCAGCAGCAGCAGCAGCAGCAGCAG-3’	
6.	eGFP-82 rc	Reverse complementary
	5’-TTGCCGTAGGTGGCATCGCCCTCGCCCTCGCCGGACACGCTGAACTTGTGGCCGTTTACG-3’	
7.	eGFP-162 rc	Reverse complementary
	5’-CCATGTGATCGCGCTTCTCGTTGGGGTCTTTGCTCAGGGCGGACTGGGTGCTCAGGTAGT-3’	
8.	Linker rc	Reverse complementary
	5’-AGGACTTGAGGGACTCGAAGGCCTTCATAGATCCGGCGGCTGAGGAAGCTGAGGAGGCGG-3’	
9.	HTT rc	Reverse complementary
	5’-CGGCGGCTGAGGAAGCTGAGAAAAAGAAGGACTTGAGGGACTCGAAGGCCTTCATAGATC-3’	
10.	HTT repeat rc	Reverse complementary
	5’-CTGCTGCTGCTGCTGCTGCTGCTGCTGCTGCTGCTGCTGCTGCTGCTGCTGCTGCTGCTG-3’	
