# Supplementary figures and images for: The dynamics of early-state transcriptional changes and aggregate formation in a Huntington’s disease cell model
Source: BMC Genomics. 2017 May 12;18:373. doi: 10.1186/s12864-017-3745-z (PMC5429582; doi:10.1186/s12864-017-3745-z)

## Slide 1
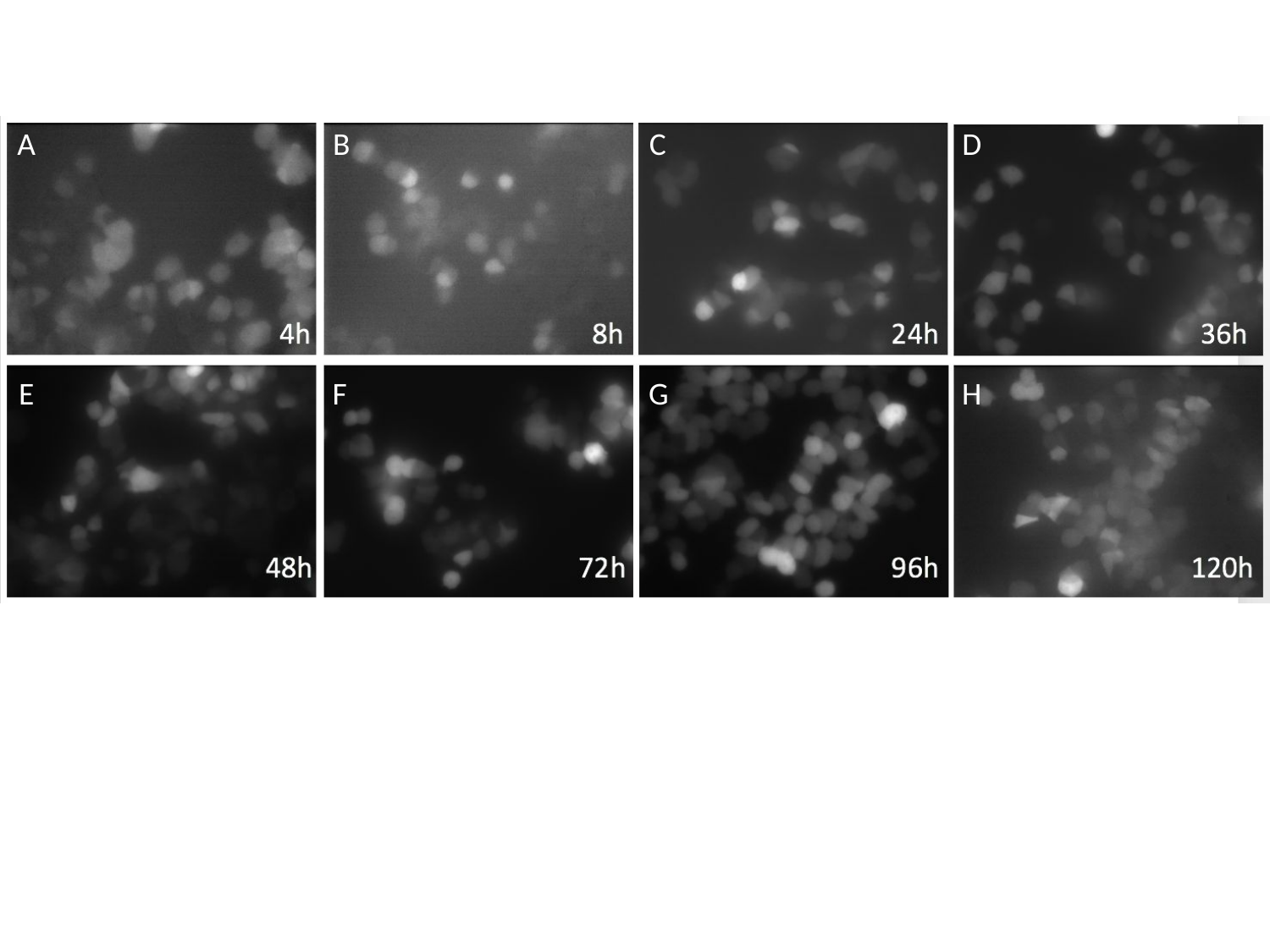

A
B
C
D
E
F
G
H

Supplement: Supplementary file 3 — Absence of eGFP-Htt-Q23 aggregate formation upon doxycycline induction. PC12 cells expressing eGFP-Htt-Q23 (control) upon doxycycline induction were imaged at various time points post-induction (4 – 120 h) using fluorescence living cell imaging. A-H show the Htt-Q23 eGFP expressing cells at the various post-induction time points (representative examples of at least four replicates per time point). (PPTX 647 kb) [file 12864_2017_3745_MOESM3_ESM.pptx]

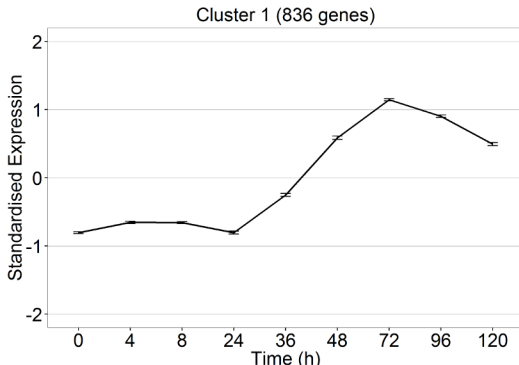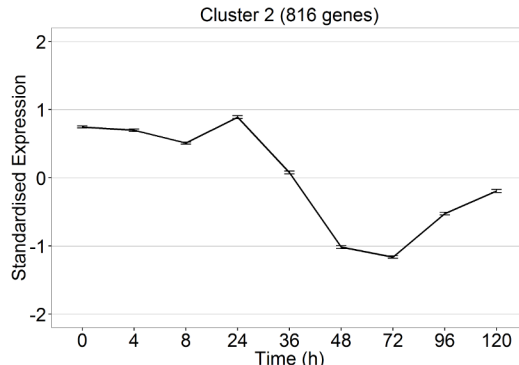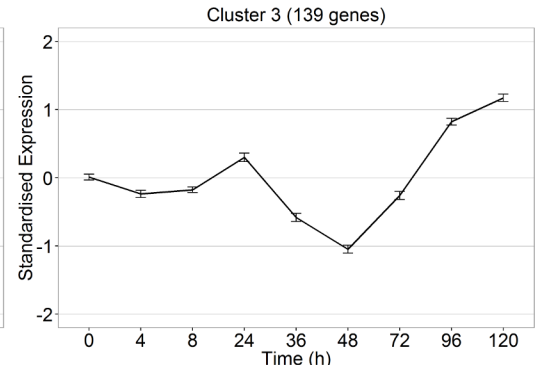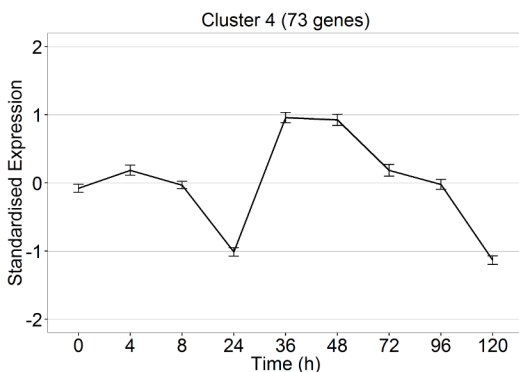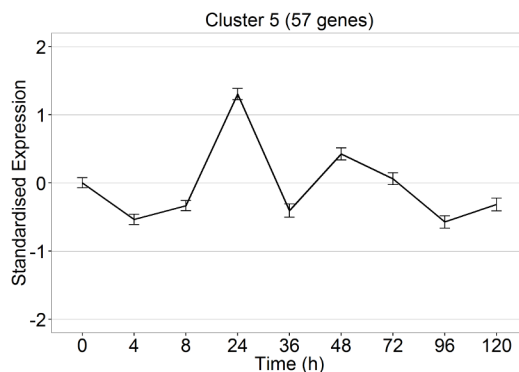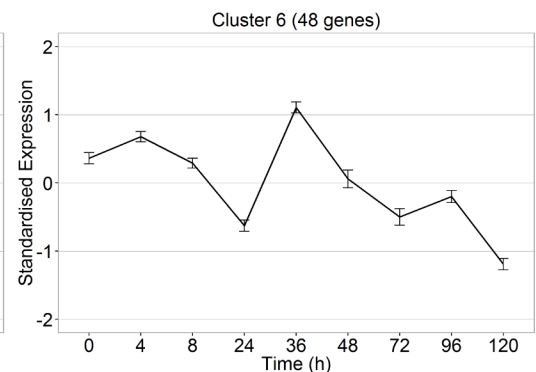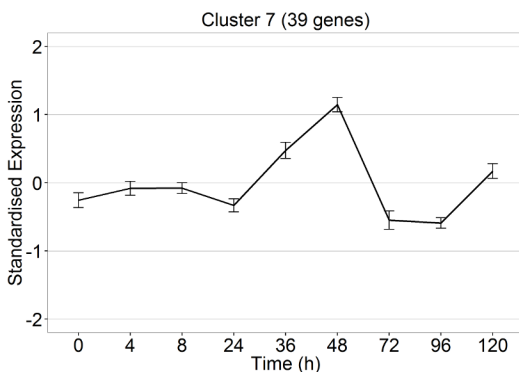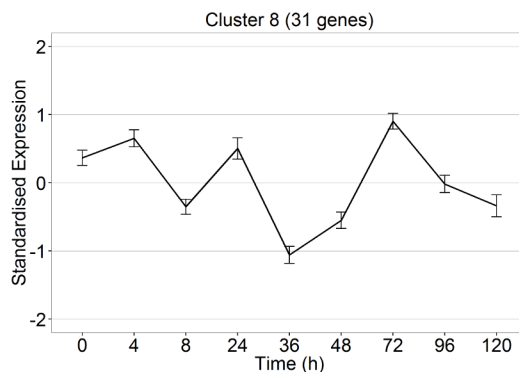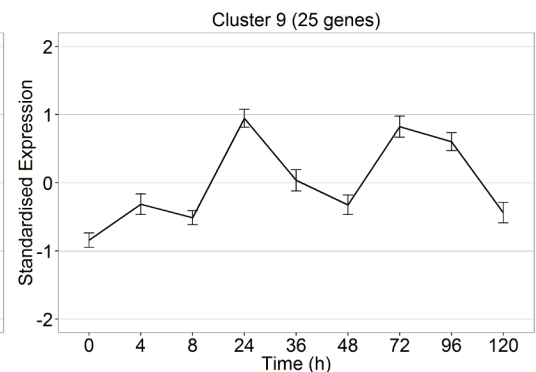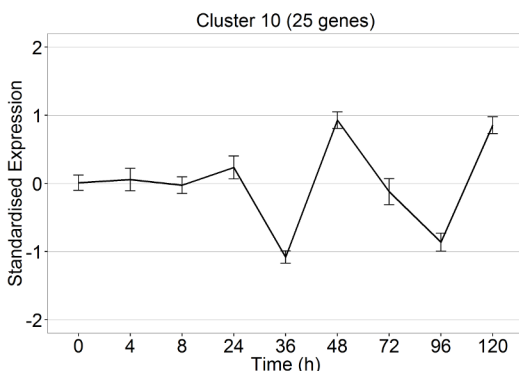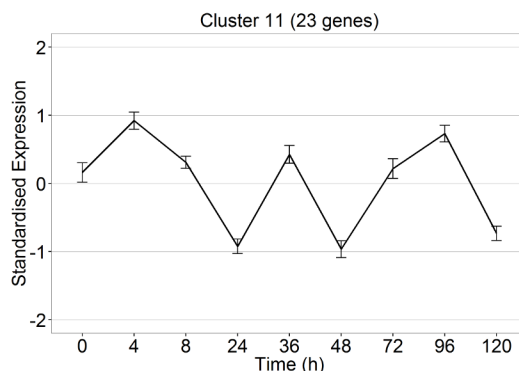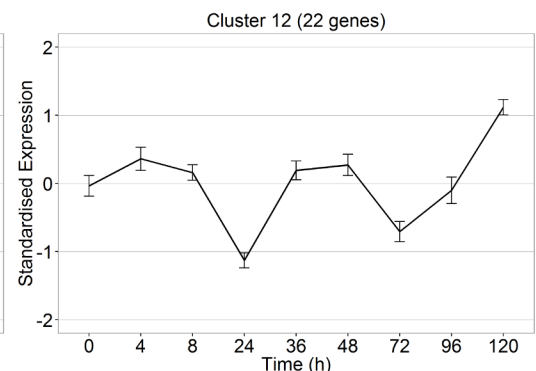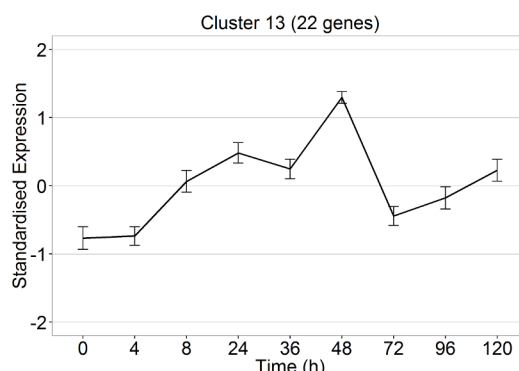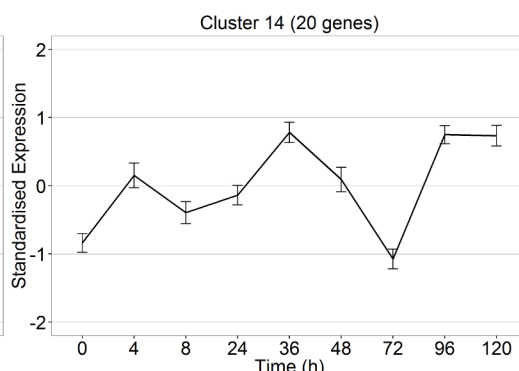

Supplement: Supplementary file 7 — Expression profiles for all fourteen clusters created using the CLICK algorithm (Expander). Expression patterns for all clusters were standardized to mean zero and standard deviation one. Error bars correspond to one standard deviation. (PDF 360 kb) [file 12864_2017_3745_MOESM7_ESM.pdf]

**A**

Cluster early persistent DE genes (101 genes)

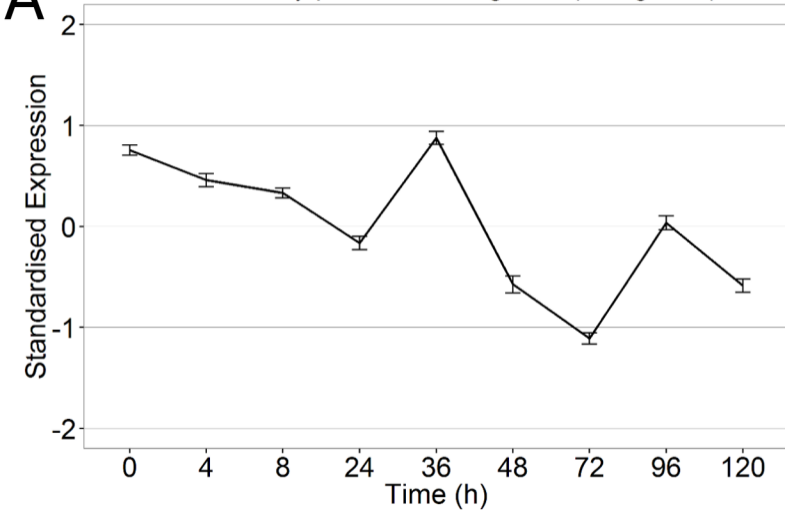**B**

Functional enrichment persistent early genes

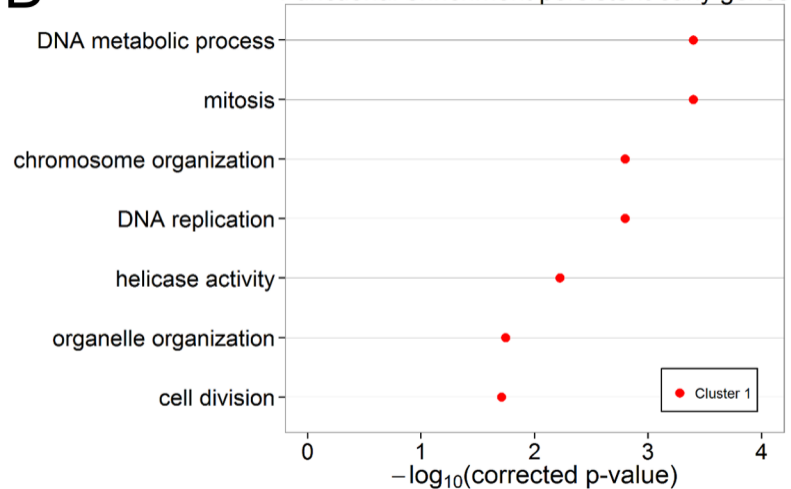

Supplement: Supplementary file 8 — Expression profile and functional enrichment analysis of early persistently DE genes. (A) Genes were selected from the HD-specific set of DE genes and further limited to those exhibiting a significant change in expression level during the full first 24 h post-induction using a relaxed significance threshold (P-value < 0.2). The resulting set of 404 genes was then subjected to cluster analysis followed by functional enrichment analysis. (B) Only one cluster was found to contain genes that overrepresented biological processes. Error bars correspond to one standard deviation. (PDF 224 kb) [file 12864_2017_3745_MOESM8_ESM.pdf]
