# Supplementary material for: The dynamics of early-state transcriptional changes and aggregate formation in a Huntington’s disease cell model
Source: BMC Genomics. 2017 May 12;18:373. doi: 10.1186/s12864-017-3745-z (PMC5429582; doi:10.1186/s12864-017-3745-z)

Symbol used in manuscript	Alias used in String	Comment
Sp1		
EGR		
CACD	?	central areolar choroidal dystrophy, not found by STRING
E2F		
UF1H3b	?	mentioned in two publications but no aliases and no NCBI entry
AP-2	TFAP2A	
HIC1		
MAZR	PATZ1	
ZF5	ZFP-161	
Pax-4		
NF-Y		
HNF4		
MAZ		
CP2/LBP-1c/LSF	TFCP2	
ZNF219		
CAC-BP	?	not found by STRING
CACCC-BF	?	not found by STRING

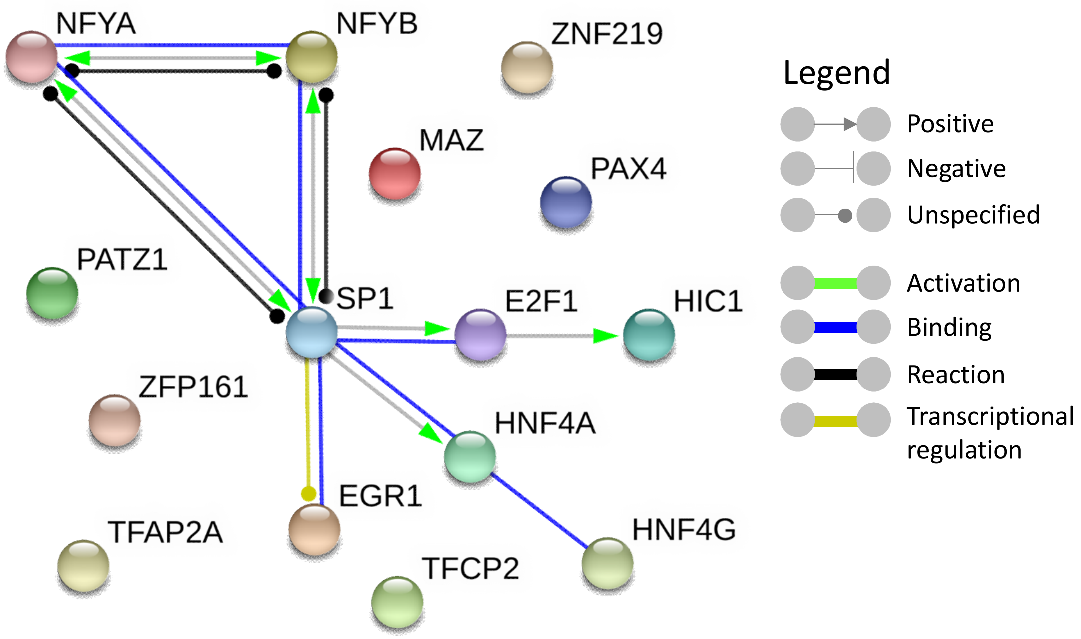

Supplement: Supplementary file 9 — Network of interactions between the transcription factors identified in the promoter analysis of DE genes in our experiment. Interactions between the transcription factors identified in the PRIMA analysis were extracted using the Search Tool for the Retrieval of Interacting Genes/Proteins (STRING) and its database of known protein interactions (http://string-db.org/; version 10.0). Gene symbols were used as input while selecting Homo sapiens as species. The network was created based on high confidence interactions (cut-off score of 0.7) and visualised using the ‘Molecular Action’ view option to show the type of interaction between the nodes. The table shows the name of the retrieved transcription factors, the alias used in STRING and a few comments. The network illustrates the detected connections. (PDF 521 kb) [file 12864_2017_3745_MOESM9_ESM.pdf]
